# Development and validation of a dynamic nomogram for acute kidney injury prediction in ICU patients with acute heart failure

**DOI:** 10.3389/fmed.2025.1544024

**Published:** 2025-02-24

**Authors:** Lu-Huai Feng, Tingting Su, Lina Huang, Tianbao Liao, Yang Lu, Lili Wu

**Affiliations:** ^1^Department of Endocrinology and Metabolism Nephrology, Guangxi Medical University Cancer Hospital, Nanning, China; ^2^Department of ECG Diagnostics, The People's Hospital of Guangxi Zhuang Autonomous Region, Nanning, China; ^3^Department of President's Office, Youjiang Medical University for Nationalities, Baise, China; ^4^Department of International Medical, Guangxi Medical University Cancer Hospital, Nanning, China

**Keywords:** acute heart failure, acute kidney injury, prediction, nomogram, intensive care unit

## Abstract

**Objective:**

Developing and validating a simple and clinically useful dynamic nomogram for predicting early acute kidney injury (AKI) in patients with acute heart failure (AHF) admitted to the intensive care unit (ICU).

**Methods:**

Clinical data from patients with AHF were obtained from the Medical Information Mart for Intensive Care IV database. The patients with AHF were randomly allocated into derivation and validation sets. The independent predictors for AKI development in AHF patients were identified through least absolute shrinkage and selection operator and multivariate logistic regression analyses. A nomogram was developed based on the results of the multivariable logistic regression to predict early AKI onset in AHF patients, which was subsequently implemented as a web-based calculator for clinical application. An evaluation of the nomogram was conducted using discrimination, calibration curves, and decision curve analyses (DCA).

**Results:**

After strict screening, 1,338 patients with AHF were included in the derivation set, and 3,129 in the validation set. Sepsis, use of human albumin, age, mechanical ventilation, aminoglycoside administration, and serum creatinine levels were identified as predictive factors for AKI in patients with AHF. The discrimination of the nomogram in both the derivation and validation sets was 0.81 (95% confidence interval: 0.78–0.83) and 0.79 (95% confidence interval: 0.76–0.83). Additionally, the calibration curve demonstrated that the predicted outcomes aligned well with the actual observations. Ultimately, the DCA curves indicated that the nomogram exhibited favorable clinical applicability.

**Conclusion:**

The nomogram that integrates clinical risk factors and enables the personalized prediction of AKI in patients with AHF upon admission to the ICU, which has the potential to assist in identifying AHF patients who would derive the greatest benefit from interventions aimed at preventing and treating AKI.

## Introduction

Acute heart failure (AHF) is a significant medical condition marked by the abrupt onset or exacerbation of symptoms and indicators of heart failure, necessitating immediate medical intervention. Given the ongoing trend of an aging population leading to a rise in hospitalizations for AHF, this condition has emerged as a prominent public health issue (1–3). It is worth noting that acute kidney injury (AKI) is very common to occur in the setting of AHF and this is referred to as cardiorenal syndrome type 1, resulting in prolonged hospital stays and high mortality rates (4, 5). There is an association between AKI and 58% of cases of congestive heart failure, and the severity of AKI can increase the risk of congestive heart failure (6). Emerging evidence indicates that cardiorenal syndrome is linked to a range of pathological mechanisms, including hemodynamics alterations, neurohormonal changes, hypervolemia, hypertension, hyperuremia, and hyperuricemia (7, 8). Given that the molecular mechanisms underlying cardiorenal syndrome have yet to be fully elucidated, no singular intervention has proven effective in its treatment (7, 9). Therefore, identifying high-risk groups for AKI and implementing targeted preventive measures are essential for improving the prognosis of patients with AHF (10). Consequently, the adoption of AKI prevention strategies is crucial for reducing mortality rates and mitigating the economic burden associated with AHF.

The current diagnostic criteria for AKI as outlined by the Kidney Disease Improving Global Outcomes (KDIGO) suggest that serum creatinine level and urinary volume are the primary clinical indicators (11). While some studies have suggested alternative biomarkers such as cystatin C, kidney injury molecule-1, neutrophil gelatinase-associated lipocalin, and liver-type fatty acid binding protein (12), for early detection of kidney damage prior to serum creatinine elevation, these biomarkers are limited in their diagnostic accuracy (12–14). As a result, further research is needed on tools that can help predict AKI in patients with AHF early.

Acute kidney injury may arise from various etiological factors, rendering early detection challenging in individuals presenting with AHF (15). The timely identification of AKI based on a singular factor is arduous. Nomograms, as predictive instruments (16), construct graphical representations derived from statistical models to aid in clinical decision-making by estimating the likelihood of a clinical event through the consideration of multiple weighted factors (17, 18). This study was conducted with the objective of developing and validating a simple and clinically useful dynamic nomogram for predicting early AKI in AHF patients admitted to the intensive care unit (ICU).

## Methods

The methodologies described in this article are consistent with the guidelines established in the Transparent Reporting of a Multivariable Prediction Model for Individual Prognosis or Diagnosis (TRIPOD) statement (19).

### Database

Study data was retrieved from the Medical Information Mart for Intensive Care *IV* (MIMIC-IV) database (version 2.2), a comprehensive critical care database that is publicly accessible and based in the United States (20). The MIMIC IV database collects clinical data from more than 190,000 patients and 450,000 hospitalizations admitted to Beth Israel Deaconess Medical Center from 2008 to 2019. The database records detailed information such as patient demographic information, laboratory tests, medication, vital signs, surgical procedures, disease diagnosis, drug management, and follow-up survival status. The Institutional Review Boards of Beth Israel Deaconess Medical Center (Boston, MA) and Massachusetts Institute of Technology (Cambridge, MA) granted approval for the establishment of the IV database, resulting in a waiver of informed consent for this study. Author L-H F completed the National Institutes of Health online training course (certification number 35897462) to access the MIMIC-IV database (version 2.2).

### Participants

Adult patients aged 18 years or older, who were admitted to the ICU with a diagnosis of acute heart failure (including both acute systolic heart failure and/or acute diastolic heart failure), based on the International Classification of Diseases (ICD), 9th or 10th revision codes, were selected from the MIMIC-IV database. This cohort includes both patients who had AHF on ICU admission and those who developed AHF during their ICU stay. Patients who had acute kidney injury prior to admission to the intensive care unit were excluded in the study. In cases where a patient had multiple admissions to the ICU, only data from their initial admission was included in the analysis.

A pre-seeded random number generator (123) in R software version 4.3.3 was employed to allocate patients into groups, which were subsequently divided into derivation and validation sets at a ratio of 7:3.

### Data extraction

Data extraction was performed using PostgreSQL tools (V.1.13.1). The following information was extracted directly or calculated using data from the database: Age, sex, laboratory variables, chronic medical conditions, comorbidities, mechanical ventilation records, the time of AKI, and administration of drugs. Laboratory variables including hemoglobin (Hb), glucose, serum creatinine (Scr), and albumin (Alb) were extracted and analyzed. Chronic medical conditions such as chronic obstructive pulmonary disease (COPD), chronic kidney disease (CKD), diabetes, chronic liver disease, and hypertension were identified in the study population. Malignancies were not identified as a comorbidity in our cohort, and therefore, were not included in the analysis. Conditions such as acute pancreatitis and sepsis, which are known to contribute to the development of acute heart failure, were documented based on recorded ICD-9 or ICD-10 codes in the MIMIC-IV database. Treatment regimens for patients consisted of vasoactive drugs, diuretics, aminoglycosides, and human albumin. Science-based, clinically important, and already-identified predictors were used to assess *a priori* risk factors for AKI (21–23).

For all laboratory test result parameters, were measured during the first 24 h in the ICU. And the use of diuretics and aminoglycosides was categorized as any administration of these medications prior to the occurrence of AKI during the ICU stay for any indication.

### Missing data handling

Within the MIMIC-IV database, the prevalence of missing data is notable. Nevertheless, the exclusion of patients with incomplete data poses a risk of introducing substantial bias into the study. To address missing data, all variables utilized in the analyses were carefully considered. It was observed that less than 10% of missing values were present across all variables. Consequently, imputation was performed by substituting missing values with means for continuous variables exhibiting normal distributions and with medians for continuous variables displaying skewed distributions (24). Furthermore, our study also did not include any missing dichotomous variables.

### Definitions and outcomes

The primary outcome was the occurrence of AKI during ICU stay. The KDGIO criteria were used to define AKI (11). Twenty-four urine output criteria to define AKI were not used because urine output was not reliably collected. Vasoactive drugs, diuretics, and aminoglycosides were classified as any instances of their administration during the patient’s ICU stay for any indication. Triglyceride glucose (TyG) index was computed using the following formula: Ln (Triglycerides [mg/dl] × Glucose [mg/dl]/2) (25).

### Statistical analysis

SPSS version 26.0 (IBM, Armonk, NY, United States) and R version 4.2.1 were used for statistical analyses. Two-sided *p*-values were used throughout, and *p* < 0.05 was considered statistically significant. A categorical variable is presented as a percentage, while a continuous variable is presented as a mean ± SD, median, or range, depending on their distribution normality. A chi-square test was used for categorical variables and a *t*-test or Wilcoxon rank sum test for continuous variables based on their distributions.

To improve both the precision of predictions and the clarity of interpretation, the study utilized least absolute shrinkage and selection operator (LASSO) regression analysis to select and regulate variables (26). The variables chosen in the LASSO regression model within the derivation set were subsequently evaluated through univariate logistic regression to assess their significance in predicting AKI (27). Variables with *p* < 0.05 in the initial univariate logistic analyses were subsequently evaluated through multivariable logistic regression utilizing a backward stepwise selection approach. Following the development of a predictive model using multivariable logistic regression, a clinical prediction nomogram and an interactive web-based application for estimating the probability of AKI were created using Shiny apps.

Nomogram performance was assessed in both the derivation and validation sets using discrimination and calibration (28). The nomogram’s discrimination was evaluated using the C-index, which varies from 0.5 (indicating no discrimination) to 1.0 (indicating perfect prediction). Calibration of the nomogram was assessed through a visual calibration plot comparing the predicted and actual probability value of AKI occurrence. Additionally, 1,000 bootstrap resamples were performed for internal validation to evaluate the predictive accuracy of the nomogram. In addition, the clinical value of the nomogram was assessed by decision curve analysis (DCA), which can determine the net benefit of predictors and models (29).

## Results

### Characteristics of patients

A total of 4,467 patients with acute heart failure was included in the study, and their characteristics are presented in Table 1. As part of the derivation set, 3,129 patients were enrolled, and 1,338 patients were included in the validation set. The incidence of AKI did not show a significant difference (*p* = 0.256) between the derivation (14%) and validation (15.3%) datasets. Despite the patients being categorized based on admission timing, their clinical characteristics and laboratory results were similar, suggesting that they could serve as both derivation and validation data.

**Table 1:** Characteristics of patients in the derivation and validation sets.

Variable	Validation set (*n* = 1,338)	Derivation set (*n* = 3,129)	*p*-value
AKI, *n*(%)			
No	1,133 (84.7)	2,692 (86.0)	0.256
Yes	205 (15.3)	437 (14.0)	
Race, *n*(%)			
White	872(65.2)	2037 (65.1)	0.762
Black	320 (23.9)	729 (23.3)	
Other	146 (10.9)	363 (11.6)	
Sex, *n*(%)			
Female	636 (47.5)	1,516 (48.4)	0.597
Male	702 (52.5)	1,613 (51.6)	
Age, years (median [IQR])	75 [64,85]	76 [64,86]	0.429
CKD, *n*(%)			
No	1,224 (91.5)	2,803 (89.6)	0.058
Yes	114 (8.5)	326 (10.4)	
COPD, *n*(%)			
No	1,315 (98.3)	3,066 (98.0)	0.591
Yes	23 (1.7)	63 (2.0)	
Coronary, *n*(%)			
No	1,240 (92.7)	2,876 (91.9)	0.421
Yes	98 (7.3)	253 (8.1)	
T2DM, *n*(%)			
No	1,216 (90.9)	2,866 (91.6)	0.472
Yes	122 (9.1)	263 (8.4)	
Hypertension, *n*(%)			
No	1,149 (85.9)	2,675 (85.5)	0.773
Yes	189 (14.1)	454 (14.5)	
Sepsis, *n*(%)			
No	1,261 (94.3)	2,964 (94.7)	0.562
Yes	77 (5.8)	165 (5.3)	
Chronic liver disease, *n*(%)			
No	1,330 (99.40)	3,113 (99.5)	0.889
Yes	8 (0.6)	16 (0.5)	
Use of human albumin, *n*(%)			
No	1,308 (97.8)	3,074 (98.2)	0.334
Yes	30 (2.2)	55 (1.8)	
Acute pancreatitis, *n*(%)			
No	1,334 (99.7)	3,127 (99.9)	0.129
Yes	4 (0.3)	2 (0.1)	
Use of diuretic, *n*(%)			
No	272 (20.3)	594 (19.0)	0.317
Yes	1,066 (79.7)	2,535 (81.0)	
Use of vasoactive drug, *n*(%)			
No	1,258 (94.0)	2,967 (94.8)	0.311
Yes	80 (6.0)	162 (5.2)	
Mechanical ventilation, *n*(%)			
No	627 (46.9)	1,525 (48.7)	0.264
Yes	711 (53.1)	1,604 (51.3)	
Use of aminoglycosides, *n*(%)			
No	1,304 (97.5)	3,065 (98.0)	0.355
Yes	34 (2.5)	64 (2.0)	
Hb, g/L (median [IQR])	104 [99, 110]	104 [98, 109]	0.847
Scr, μmmol/L(median [IQR])	123.8 [88.4, 187.9]	123.8 [97.2, 176.8]	0.423
BUN, mmol/L(median [IQR])	11.1 [7.5, 18.2]	10.7 [7.1, 16.8]	0.085
Alb, g/L (median [IQR])	38.0 [33.0, 41.0]	38.0 [34.0, 41.0]	0.261
TyG index (median [IQR])	8.8 [8.3, 9.2]	8.8 [8.3, 9.3]	0.012
Lactate, mmol/L	2.3(1.7,2.9)	2.1(1.6,2.9)	0.135

AKI, acute kidney injury; COPD, chronic obstructive pulmonary disease; CKD, chronic kidney disease; ALB, albumin; BUN, Blood urea nitrogen; TyG index, triglyceride glucose index.

### Model development and specifications of AKI

**Figure 1:**
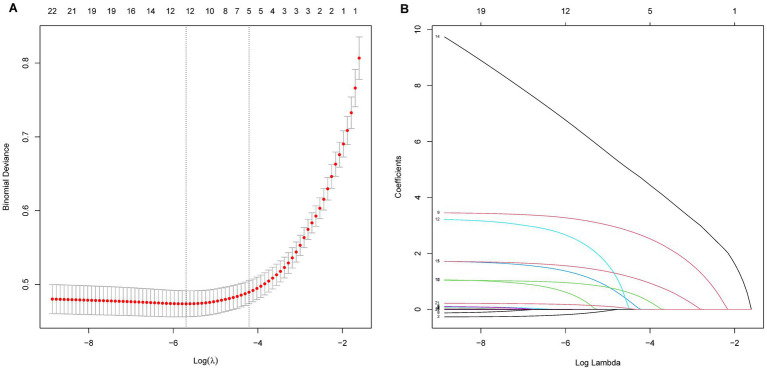
LASSO regression analysis for variables selection in predicting AKI in AHF patients. Panel **A** shows the deviance (binomial) as a function of the log of the regularization parameter λ. The red dots represent the deviance at each λ value, with vertical dotted lines indicating the selected λ values based on cross-validation. Panel **B** illustrates the evolution of coefficients as a function of the log of λ, with each line representing a feature. As λ increases, the coefficients shrink, with some becoming exactly zero, indicating feature selection.

The LASSO regression analysis was employed to simplify the model by reducing the number of features from 22 to 12 potential predictors within the derivation set, as depicted in Figures 1A,B. The predictors linked to AKI identified by the LASSO regression method are outlined in Table 2 (lambda = 0.003353464). Subsequently, a multivariable logistic regression analysis was conducted to further investigate the variables that passed through both univariate logistic regression and LASSO analyses.

**Table 2:** Univariate and multivariate logistic regression analyses of variables relating to AKI in the derivation set.

Variable	Univariate analysis		Multivariate analysis	
	OR (95% CI)	*P*-value	OR (95% CI)	*P*-value
Sex				
Female	Reference	0.858		
Male	1.02(0.83,1.25)			
Sepsis				
No	Reference	<0.001	Reference	<0.001
Yes	19.4(13.7,27.8)		28.5(19.0,42.8)	
Chronic liver disease				
No	Reference	0.581		
Yes	1.4(0.4,5.0)			
Use of human albumin				
No	Reference	<0.001	Reference	<0.001
Yes	16.4(9.1,29.6)		15.3(7.6,30.4)	
Acute pancreatitis				
No	Reference	0.198		
Yes	6.2(0.4,98.9)			
Use of vasoactive drug				
No	Reference	0.552		
Yes	831410.0(0, inf)			
Mechanical ventilation				
No	Reference	<0.001	Reference	<0.001
Yes	4.5(3.5,5.7)		5.1(3.8,6.8)	
Use of aminoglycosides				
No	Reference	<0.001	Reference	<0.001
Yes	7.5(4.5,12.4)		4.0(2.2,7.3)	
Scr	1.1(1.1,1.2)	<0.001	1.1(1.0,1.2)	<0.001
BUN	1.2(1.0,1.3)	0.008	1.1(0.9,1.2)	0.363
Alb	0.8(0.7,0.9)	<0.001	0.9(0.8,1.0)	0.166
TyG index	1.2(1.0,1.4)	0.008	1.1(1.0,1.3)	0.114

**Figure 2:**
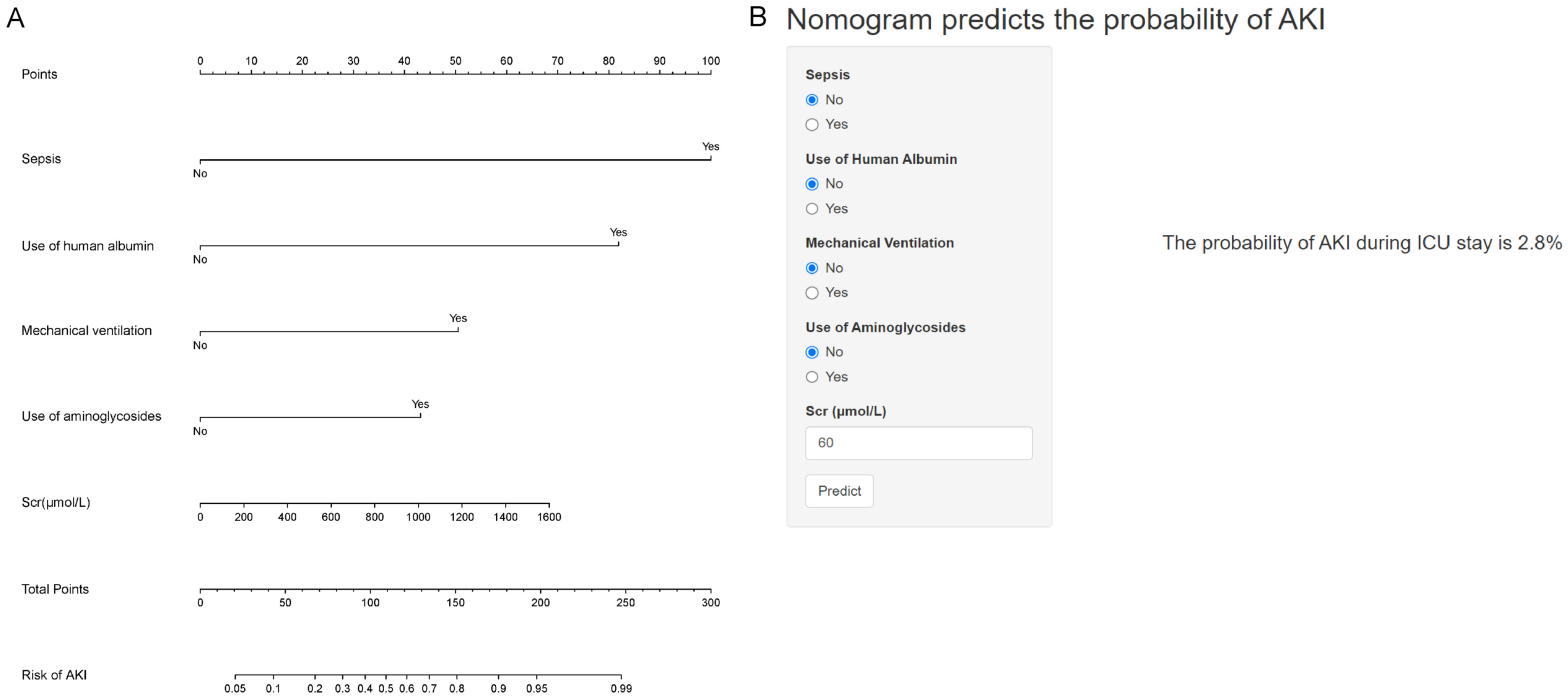
Nomogram for predicting the probability of AKI in AHF patients. **(A)** Nomogram for estimating the risk of AKI. Points are assigned for each variable, including sepsis, use of human albumin, mechanical ventilation, use of aminoglycosides, and serum creatinine (Scr) levels. The total points correspond to the probability of AKI. **(B)** An example of using the nomogram: input values for sepsis (No), use of human albumin (No), mechanical ventilation (No), use of aminoglycosides (No), and Scr (60 μmol/L). The predicted probability of AKI during ICU stay is 2.8%.

The ultimate multivariable logistic model revealed five predictors (sepsis, use of human albumin, mechanical ventilation, use of aminoglycosides, and Scr levels measured in μmol/L). The nomogram (Figure 2A) illustrates the model that integrates independent predictors and is accessible online^1^, as depicted in a screenshot in Figure 2B. To utilize the interactive online nomogram, users are required to choose between “Yes” or “No” in the respective options, input the relevant laboratory test results, and subsequently select “Predict” to ascertain the likelihood of AKI occurrence during patients’ ICU admission.

### Nomogram performance in the derivation set

**Figure 3:**
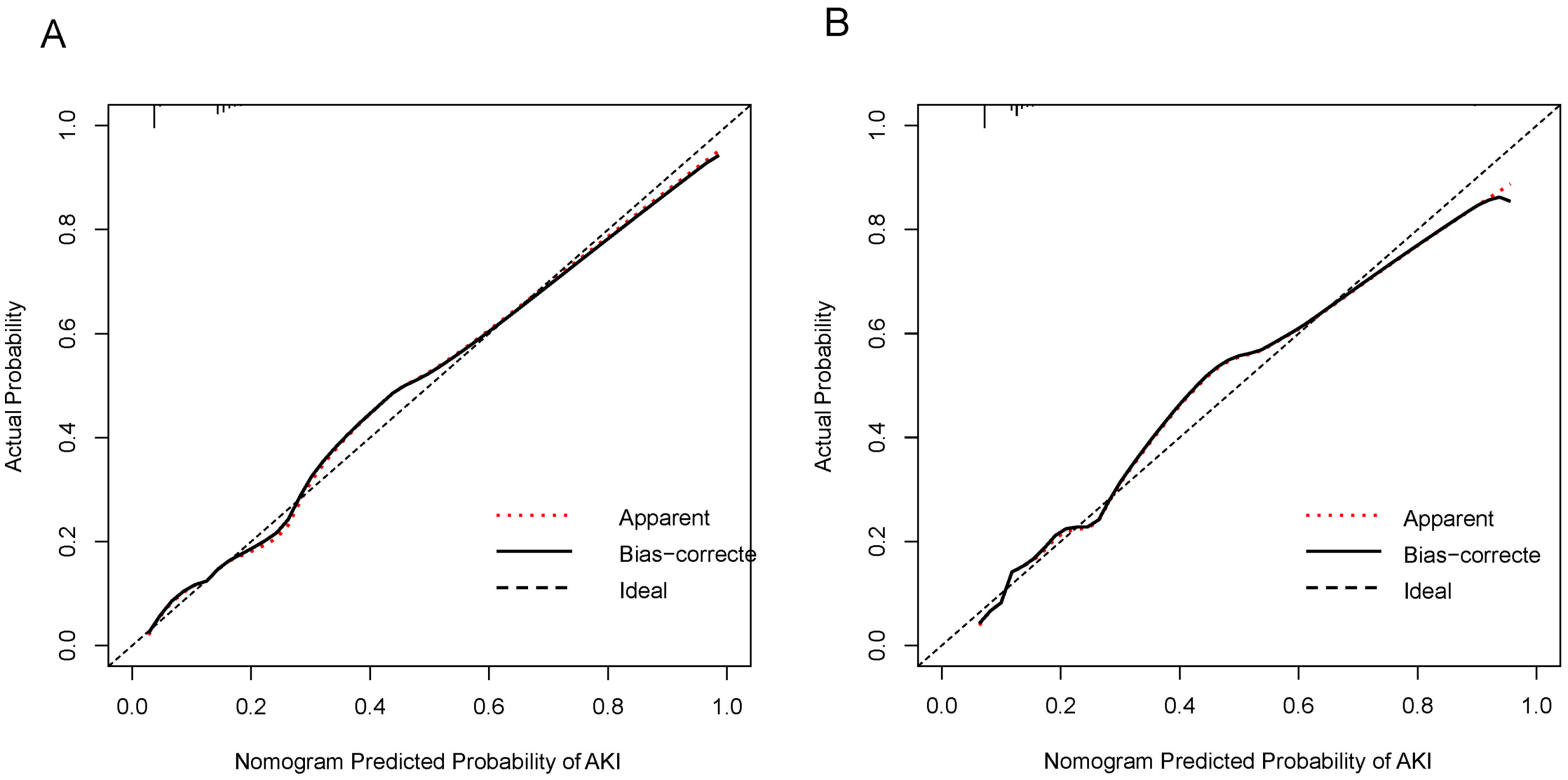
Calibration curves for the nomogram predicting AKI in AHF patients. **(A)** Calibration curve in the derivation set. The *x*-axis represents the nomogram-predicted probability of AKI, and the *y*-axis represents the actual probability of AKI. The diagonal dashed line represents a perfect prediction by an ideal model. The solid line shows the performance of the nomogram, where a closer fit to the diagonal line indicates better predictive accuracy. The dotted line indicates apparent predictions without bias correction. **(B)** Calibration curve in the validation set. The axes and lines are the same as in **(A)**. The solid line represents the bias-corrected performance of the nomogram, showing the agreement between predicted and actual outcomes in the validation cohort.

The C-index of the prediction nomogram was determined to be 0.81 [95% confidence interval (CI): 0.78–0.83] for the derivation set. The calibration curve depicted in Figure 3A demonstrates a favorable alignment between the predicted and observed outcomes for the probability of AKI in the derivation set. The non-significant result (*p* = 0.540) of the Hosmer–Lemeshow test suggests that the model did not exhibit over-fitting.

### Nomogram performance in the validation set

In the validation set, the nomogram demonstrated a C-index of 0.79 (95%CI: 0.76–0.83) for predicting AKI. Additionally, the calibration curve (Figure 3B) indicated acceptable consistency like the derivation set, between the observed and nomogram-predicted. Probabilities of AKI.

### Clinical use of nomogram

**Figure 4:**
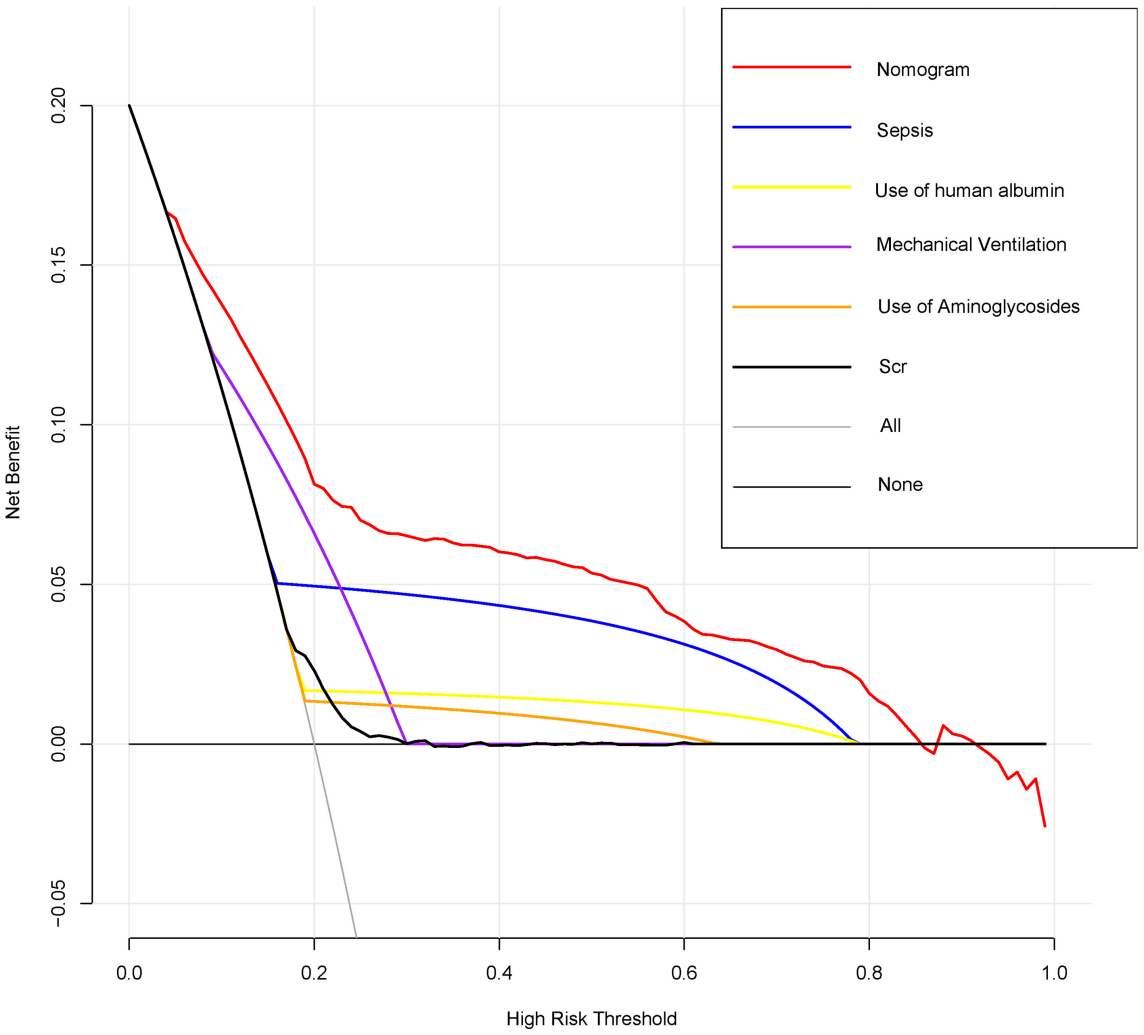
Decision curve analysis for the nomogram predicting AKI in AHF patients. Decision curve analysis comparing the net benefits of the nomogram and individual predictors across a range of high-risk thresholds. The *x*-axis represents the high-risk threshold for predicting AKI, and the *y*-axis represents the net benefit. The red line represents the nomogram, showing the highest net benefit across most thresholds compared to individual predictors such as sepsis, use of human albumin, mechanical ventilation, use of aminoglycosides, and Scr.

Figure 4 displays the results of decision curve analysis for the nomogram, indicating the high-risk threshold probability at which a clinician may determine a patient’s risk of AKI and potential benefit from intervention. The decision curve suggests that utilizing the nomogram for AKI prediction can provide significant benefit when a clinician’s threshold probability >5%, with the nomogram demonstrating superior predictive power compared to a single predictor within this range.

## Discussion

We have created and confirmed the accuracy of a nomogram designed to forecast the likelihood of AKI in ICU patients with AFH through a straightforward and feasible method. This nomogram, which includes variables such as sepsis, use of human albumin, mechanical ventilation, use of aminoglycosides, and Scr levels, can predict the occurrence of AKI in AHF patients upon their admission to the ICU, aligning with the current emphasis on personalized medicine. Our model demonstrated consistent performance in both the derivation and validation groups, showing strong discrimination and calibration capabilities. When comparing the approach of categorizing all patients as either non-AKI or AKI, the nomogram demonstrates superior clinical utility by yielding a net benefit exceeding that of any individual factor for a risk threshold greater than 5%.

As the kidneys are particularly sensitive to abrupt changes in cardiac output, acute renal hypoperfusion results in a decrease in glomerular filtration rate, urine output, and parenchymal oxygenation, resulting in acute kidney injury. Therefore, on the basis of acute heart failure, any factor affecting renal hypoperfusion or renal parenchymal damage increases the risk of AKI. The circulatory system links the heart and kidneys, meaning that primary dysfunction in one often leads to secondary dysfunction or damage in the other (7). Oxidative stress, inflammation, and an overactive renin-angiotensin-aldosterone system are key contributors to cardiorenal syndrome. Heart-kidney interactions are often influenced by factors such as obesity, metabolic syndrome, cachexia, diabetes, hypertension, proteinuria, uremia, and anemia, which predispose individuals to AKI and heart failure (7, 8). Moreover, sepsis, a well-established cause of AKI, further complicates this interaction. In sepsis, the kidney is often one of the first organs affected, with up to two-thirds of septic shock patients developing AKI (30). Here, microcirculatory dysfunction and systemic inflammation are pivotal in driving AKI, highlighting the intricate relationship between heart and kidney dysfunction in critically ill patients (31).

Albumin combined with diuretics (such as furosemide) is a common treatment for acute heart failure (32), however, there is insufficient evidence to support widespread use of furosemide and albumin in clinical practice for the benefit of patients with acute heart failure (32, 33). In individuals presenting with AHF and fluid overload, diuretic therapy aimed at decongestion often results in elevated serum creatinine levels and AKI. Nonetheless, in the long term, effective decongestion is associated with improved survival rates and a reduction in hospital admissions, despite the initial increase in serum creatinine and occurrence of AKI (34). Additionally, tolvaptan has been shown to decrease the incidence of AKI in patients with acute decompensated heart failure and advanced chronic kidney disease (35).The association between the administration of human blood albumin and the incidence of acute kidney injury is a topic of debate (36, 37). Our study found a positive correlation between albumin use and the risk of AKI in individuals with acute heart failure. However, it is important to note that albumin is typically administered to more critically ill patients, and it is possible that the observed association reflects the underlying severity of the patients’ condition rather than a direct effect of albumin itself. In other words, sicker patients may be more likely to receive albumin, and it is this illness severity, rather than the albumin administration itself, that may contribute to the higher risk of AKI.

Additionally, while our model highlights Scr as a predictor, we acknowledge that the lower creatinine values in some patients appear counterintuitive, as patients with CKD are typically more susceptible to AKI due to impaired autoregulation. The role of creatinine in AKI prediction may therefore be more complex and warrants further investigation. Furthermore, the use of mechanical ventilation, which we also found to be associated with AKI, can influence AKI risk through several mechanisms, including changes in hemodynamics, alterations in neurohormonal levels, and induction of systemic inflammation (38, 39), a number of studies have shown that mechanical ventilation is associated with the occurrence of AKI in intensive care unit patients (40–42), and our study also obtained similar results. Heart failure often involves systemic inflammation, with immune dysregulation and oxidative stress being a key factor in its progression and AKI development (9). These processes play a crucial role in cardiorenal interactions, especially concerning inflammation-induced tubular damage and microcirculatory disturbances. Research indicates that the SH2D2A gene is instrumental in regulating T cell activation and immune response through the T cell-specific adaptor protein it encodes (43), this gene is implicated in the activation of inflammatory pathways, including the NF-κB signaling pathway and cytokine regulation. Dysfunction in SH2D2A may elevate the risk of AKI in the context of heart failure, underscoring the necessity to investigate its mechanisms for the development of targeted therapeutic interventions. It is no doubt that aminoglycoside antibiotics are associated with the development of acute kidney injury as a result of renal toxicity. The administration of aminoglycoside antibiotics to patients with acute heart failure represents a high-risk population for AKI. Our findings further demonstrate a positive correlation between the use of aminoglycoside antibiotics and the risk of AKI. Consequently, a nomogram was constructed incorporating sepsis, human albumin administration, mechanical ventilation, and aminoglycoside usage, utilizing serum creatinine levels as a predictor for assessing the likelihood of AKI in individuals with AHF. This nomogram addresses the issue of inadequate sensitivity of serum creatinine and offers a foundation for early AKI intervention in AHF patients.

Stratified AKI risk management in patients with AHF is necessary because of the wide variation in patient outcomes. The Forman risk score (44), originally introduced in 2004 with a focus on hospitalized heart failure patients, underwent external validation in AHF patients and has since become widely recognized as a prominent predictive model on a global scale. Subsequent to the development of the Basel risk score, additional prediction models were introduced by Wang et al. and Zhou et al. between 2011 and 2016 (45–47). Nevertheless, variations in the definition of AKI or worsening renal function are evident across these studies, attributable to changes in AKI classification from the RIFLE and AKIN criteria to the KDIGO guidelines in recent years (48). Therefore, most of the models are no longer applicable to the current AKI diagnostic criteria, and it is necessary to develop a new clinical prediction model based on the current AKI diagnostic criteria to provide personalized treatment for patients with AHF. While the AKI prediction model created by Wang et al. adheres to KIDGO’s diagnostic criteria for AKI (46), it is limited by its reliance on manual calculation methods. In contrast, our model offers automated risk value calculations for AKI upon input of relevant variables, streamlining the process and enhancing the efficiency of clinical services. Preoperatively, physicians and patients can use this easy-to-use scoring system to predict their risk of AKI, a move in line with personalized medicine’s current trend (49).

The primary and concluding rationale for utilizing the nomogram lies in the necessity to assess the specific requirements for additional treatment or care on an individual basis. Nevertheless, the predictive accuracy, discriminatory ability, and calibration of the nomogram may not fully encompass the clinical implications of a given level of discrimination or extent of miscalibration (50–52). Hence, in order to validate the clinical utility of our nomogram, we conducted an evaluation to determine if decisions aided by the nomogram would lead to enhanced patient outcomes. Given the challenges associated with conducting a multi-institutional prospective validation due to the complexities of collecting clinical data from various institutions, decision curve analysis was employed as an alternative approach in this study. This innovative methodology provides a means to assess the clinical implications of decisions based on threshold probability, ultimately allowing for the calculation of net benefit (49, 53). The decision curve analysis in this study demonstrates that utilizing a nomogram for predicting AKI is more beneficial when the threshold probability for patients or physicians exceeds 5%, compared to either treating all patients or treating none.

There are certain limitations to this study. First, this study is conducted in a monocentric manner within a single ICU, despite its large cohort size. As a result, the generalizability of the findings and the dynamic online nomogram could be limited in Europe and other settings different from US. Further research is necessary to validate this model in diverse settings. Second, due to the absence of novel biomarkers, including cystatin C, neutral gelatin-associated lipocalcin, N-terminal pro B-type natriuretic peptide, urinary neutrophil gelatinase-associated lipocalin, and urinary angiotensinogen, in the MIMIC database, we were unable to enhance the predictive capacity of the model and substantiate the evaluation of current models. Third, due to the retrospective nature of the study, inherent biases such as selection bias and confounding factors cannot be fully eliminated. While stringent inclusion criteria were implemented to ensure that both the control and case groups accurately represented real-world conditions, potential biases still exist, particularly in the selection of medications (e.g., aminoglycosides) and conditions like sepsis, which may not be representative of all clinical settings. We acknowledge these biases and recommend further prospective studies to confirm the robustness of the model. Finally, a key limitation of this study is that it does not reflect the severity of acute heart failure, which is an important factor in the development of AKI. Clinicians often consider the severity of heart failure when managing AKI, as more severe heart failure is associated with a higher likelihood of developing AKI. Unfortunately, the MIMIC database does not include comprehensive information on heart failure severity, and thus, this limitation should be considered when interpreting our findings.

## Conclusion

Our research introduces a novel online nomogram that integrates clinical risk factors and enables the personalized prediction of AKI in patients with AHF upon admission to the ICU. This tool has the potential to assist in identifying AHF patients who would derive the greatest benefit from interventions aimed at preventing and treating AKI.

## Data Availability

The raw data supporting the conclusions of this article will be made available by the authors, without undue reservation.

## Footnotes

^1^ https://dynamicnomogram6666.shinyapps.io/Acute-heart-failure/

## References

[1] Dai Z Zhang Y Ye H Zhang G Jin H Chen Z. Adiponectin is valuable in the diagnosis of acute heart failure with renal insufficiency. Exp Ther Med. (2018) 16:2725–34. doi: 10.3892/etm.2018.6511, PMID: 30210613 PMC6122544

[2] Tsao CW Aday AW Almarzooq ZI Anderson CAM Arora P Avery CL. Heart disease and stroke Statistics-2023 update: a report from the American Heart Association. Circulation. (2023) 147:e93–e621. doi: 10.1161/cir.0000000000001123, PMID: 36695182 PMC12135016

[3] Mozaffarian D Benjamin EJ Go AS Arnett DK Blaha MJ Cushman M. Heart disease and stroke statistics--2015 update: a report from the American Heart Association. Circulation. (2015) 131:e29–e322. doi: 10.1161/cir.0000000000000152 25520374

[4] Prins KW Thenappan T Markowitz JS Pritzker MR. Cardiorenal syndrome type 1: renal dysfunction in acute decompensated heart failure. J Clin Outcomes Manag. (2015) 22:443–54. PMID: 27158218 PMC4855293

[5] Cosentino N Genovese S Campodonico J Bonomi A Lucci C Milazzo V. High-sensitivity C-reactive protein and acute kidney injury in patients with acute myocardial infarction: a prospective observational study. J Clin Med. (2019) 8:2192. doi: 10.3390/jcm8122192, PMID: 31842300 PMC6947188

[6] Go AS Hsu CY Yang J Tan TC Zheng S Ordonez JD. Acute kidney injury and risk of heart failure and atherosclerotic events. Clin J Am Soc Nephrol. (2018) 13:833–41. doi: 10.2215/cjn.12591117, PMID: 29773712 PMC5989674

[7] Zhao BR Hu XR Wang WD Zhou Y. Cardiorenal syndrome: clinical diagnosis, molecular mechanisms and therapeutic strategies. Acta Pharmacol Sin. (2025). doi: 10.1038/s41401-025-01476-z PMC12098865 39910210

[8] Ronco C Bellasi A Di Lullo L. Implication of acute kidney injury in heart failure. Heart Fail Clin. (2019) 15:463–76. doi: 10.1016/j.hfc.2019.05.002, PMID: 31472882

[9] Tasić D Dimitrijević Z. The role of oxidative stress as a mechanism in the pathogenesis of acute heart failure in acute kidney. Injury. (2024) 14:43. doi: 10.3390/diagnostics14182094, PMID: 39335773 PMC11431490

[10] Gudsoorkar PS Thakar CV. Acute kidney injury, heart failure, and health outcomes. Cardiol Clin. (2019) 37:297–305. doi: 10.1016/j.ccl.2019.04.005, PMID: 31279423

[11] Kellum JA Lameire N. Diagnosis, evaluation, and Management of Acute Kidney Injury: a Kdigo summary (part 1). Crit Care. (2013) 17:204. doi: 10.1186/cc11454, PMID: 23394211 PMC4057151

[12] Ronco C Bellomo R Kellum JA. Acute kidney injury. Lancet. (2019) 394:1949–64. Epub 2019/11/30. doi: 10.1016/s0140-6736(19)32563-2 31777389

[13] Bonavia A Singbartl K. Kidney injury and electrolyte abnormalities in liver failure. Semin Respir Crit Care Med. (2018) 39:556–65. doi: 10.1055/s-0038-1673616, PMID: 30485886

[14] Angeli P Gines P Wong F Bernardi M Boyer TD Gerbes A. Diagnosis and Management of Acute Kidney Injury in patients with cirrhosis: revised consensus recommendations of the International Club of Ascites. Gut. (2015) 64:531–7. doi: 10.1136/gutjnl-2014-308874, PMID: 25631669

[15] Gaspar A Iturricha-Cáceres MF Macedo E Mehta RL Claure-Del GR. The use of a medical application improves the diagnosis of acute kidney injury: a pre-post study. Front Med. (2022) 9:817387. doi: 10.3389/fmed.2022.817387, PMID: 36052325 PMC9426674

[16] Iasonos A Schrag D Raj GV Panageas KS. How to build and interpret a nomogram for Cancer prognosis. J Clin Oncol. (2008) 26:1364–70. doi: 10.1200/jco.2007.12.9791, PMID: 18323559

[17] Cho JK Lee GJ Yi KI Cho KS Choi N Kim JS. Development and external validation of nomograms predictive of response to radiation therapy and overall survival in nasopharyngeal Cancer patients. Eur J Cancer. (2015) 51:1303–11. doi: 10.1016/j.ejca.2015.04.003, PMID: 25934438

[18] Oearsakul T Tunthanathip T. Development of a nomogram to predict the outcome of moderate or severe pediatric traumatic brain injury. Turk J Emerg Med. (2022) 22:15–22. doi: 10.4103/2452-2473.336107, PMID: 35284689 PMC8862794

[19] Collins GS Reitsma JB Altman DG Moons KG. Transparent reporting of a multivariable prediction model for individual prognosis or diagnosis (Tripod): the Tripod statement. The tripod group. Circulation. (2015) 131:211–9. doi: 10.1161/circulationaha.114.014508, PMID: 25561516 PMC4297220

[20] Johnson A Bulgarelli L Pollard T Horng S Celi LA Mark R. Mimic-iv (version 2.2). Physionet. (2023). doi: 10.13026/6mm1-Ek67

[21] Liu J Xie H Ye Z Li F Wang L. Rates, predictors, and mortality of Sepsis-associated acute kidney injury: a systematic review and Meta-analysis. BMC Nephrol. (2020) 21:318. doi: 10.1186/s12882-020-01974-8, PMID: 32736541 PMC7393862

[22] Bellomo R Kellum JA Ronco C Wald R Martensson J Maiden M. Acute kidney injury in Sepsis. Intensive Care Med. (2017) 43:816–28. doi: 10.1007/s00134-017-4755-7, PMID: 28364303

[23] Zhou J Bai Y Wang X Yang J Fu P Cai D. A simple risk score for prediction of Sepsis associated-acute kidney injury in critically ill patients. J Nephrol. (2019) 32:947–56. doi: 10.1007/s40620-019-00625-y, PMID: 31313123

[24] Zhang Z. Missing data imputation: focusing on single imputation. Ann Trans Med. (2016) 4:9. doi: 10.3978/j.issn.2305-5839.2015.12.38, PMID: 26855945 PMC4716933

[25] Jiang Y Chen P Zhao Y Cai J Liang J Cheng S. Association between triglyceride glucose index and all-cause mortality in patients with cerebrovascular disease: a retrospective study. Diabetol Metab Syndr. (2024) 16:1. doi: 10.1186/s13098-023-01243-2, PMID: 38173012 PMC10763128

[26] Sauerbrei W Royston P Binder H. Selection of important variables and determination of functional form for continuous predictors in multivariable model building. Stat Med. (2007) 26:5512–28. doi: 10.1002/sim.3148, PMID: 18058845

[27] Friedman J Hastie T Tibshirani R. Regularization paths for generalized linear models via coordinate descent. J Stat Softw. (2010) 33:1–22. doi: 10.18637/jss.v033.i01, PMID: 20808728 PMC2929880

[28] Alba AC Agoritsas T Walsh M Hanna S Iorio A Devereaux PJ. Discrimination and calibration of clinical prediction models: Users' guides to the medical literature. JAMA. (2017) 318:1377–84. doi: 10.1001/jama.2017.12126, PMID: 29049590

[29] Vickers AJ Elkin EB. Decision curve analysis: a novel method for evaluating prediction models. Med Decis Making. (2006) 26:565–74. doi: 10.1177/0272989x06295361, PMID: 17099194 PMC2577036

[30] Hoste EA Bagshaw SM Bellomo R Cely CM Colman R Cruz DN. Epidemiology of acute kidney injury in critically ill patients: the multinational Aki-epi study. Intensive Care Med. (2015) 41:1411–23. doi: 10.1007/s00134-015-3934-7, PMID: 26162677

[31] Manrique-Caballero CL Del Rio-Pertuz G Gomez H. Sepsis-associated acute kidney injury. Crit Care Clin. (2021) 37:279–301. doi: 10.1016/j.ccc.2020.11.010, PMID: 33752856 PMC7995616

[32] Mullens W Damman K Harjola VP Mebazaa A Brunner-La Rocca HP Martens P. The use of diuretics in heart failure with congestion – a position statement from the heart failure Association of the European Society of cardiology. Eur J Heart Fail. (2019) 21:137–55. doi: 10.1002/ejhf.1369 30600580

[33] Fernandes J Costa R Guerreiro R Bonifácio D Rodrigues A Henriques C. Co-Administration of Albumin and Furosemide in acute heart failure with diuretics resistance. Acta Medica Port. (2023) 36:193–201. doi: 10.20344/amp.17714, PMID: 36762993

[34] Banerjee D Ali MA Wang AY Jha V. Acute kidney injury in acute heart failure-when to worry and when not to worry? Nephrol Dial Transplant. (2024) 40:10–8. doi: 10.1093/ndt/gfae146, PMID: 38944413 PMC11879425

[35] Tagaya T Hayashi H Ogata S Takahashi K Koide S Inaguma D. Tolvaptan's association with low risk of acute kidney injury in patients with advanced chronic kidney disease and acute decompensated heart failure. Am J Nephrol. (2023) 54:319–28. doi: 10.1159/000531692, PMID: 37385233

[36] Frenette AJ Bouchard J Bernier P Charbonneau A Nguyen LT Rioux JP. Albumin administration is associated with acute kidney injury in cardiac surgery: a propensity score analysis. Crit Care. (2014) 18:602. doi: 10.1186/s13054-014-0602-1, PMID: 25394836 PMC4256900

[37] Lee EH Kim WJ Kim JY Chin JH Choi DK Sim JY. Effect of exogenous albumin on the incidence of postoperative acute kidney injury in patients undergoing off-pump coronary artery bypass surgery with a preoperative albumin level of less than 4.0 G/dl. Anesthesiology. (2016) 124:1001–11. doi: 10.1097/aln.0000000000001051, PMID: 26891150

[38] Visconti L Santoro D Cernaro V Buemi M Lacquaniti A. Kidney-lung connections in acute and chronic diseases: current perspectives. J Nephrol. (2016) 29:341–8. doi: 10.1007/s40620-016-0276-7, PMID: 26940339

[39] Hepokoski ML Malhotra A Singh P Crotty Alexander LE. Ventilator-induced kidney injury: are novel biomarkers the key to prevention? Nephron. (2018) 140:90–3. doi: 10.1159/000491557, PMID: 29996132 PMC6165693

[40] Sitina M Sramek V Helan M Suk P. Prognostic significance of early acute kidney injury in Covid-19 patients requiring mechanical ventilation: a single-center retrospective analysis. Ren Fail. (2023) 45:2205954. doi: 10.1080/0886022x.2023.2205954, PMID: 37133859 PMC10158536

[41] Tsigkou V Siasos G Oikonomou E Bletsa E Vavuranakis M Tousoulis D. “Heart failure in COVID-19 patients: critical care experience”: a letter to the editor. World J Virol. (2022) 11:216–20. doi: 10.5501/wjv.v11.i4.216, PMID: 36159614 PMC9372782

[42] Husain-Syed F Birk HW Seeger W Ronco C. A protective kidney-lung approach to improve outcomes in mechanically ventilated patients. Blood Purif. (2016) 42:214–8. doi: 10.1159/000448471, PMID: 27522219

[43] Wedel J Stack MP Seto T Sheehan MM Flynn EA Stillman IE. T cell-specific adaptor protein regulates mitochondrial function and Cd4(+) T regulatory cell activity in vivo following transplantation. J Immunol. (2019) 203:2328–38. doi: 10.4049/jimmunol.1801604, PMID: 31541025 PMC6783373

[44] Forman DE Butler J Wang Y Abraham WT O'Connor CM Gottlieb SS. Incidence, predictors at admission, and impact of worsening renal function among patients hospitalized with heart failure. J Am Coll Cardiol. (2004) 43:61–7. doi: 10.1016/j.jacc.2003.07.031, PMID: 14715185

[45] Breidthardt T Socrates T Noveanu M Klima T Heinisch C Reichlin T. Effect and clinical prediction of worsening renal function in acute decompensated heart failure. Am J Cardiol. (2011) 107:730–5. doi: 10.1016/j.amjcard.2010.10.056, PMID: 21247523

[46] Wang YN Cheng H Yue T Chen YP. Derivation and validation of a prediction score for acute kidney injury in patients hospitalized with acute heart failure in a Chinese cohort. Nephrology. (2013) 18:489–96. doi: 10.1111/nep.12092, PMID: 23607443

[47] Zhou LZ Yang XB Guan Y Xu X Tan MT Hou FF. Development and validation of a risk score for prediction of acute kidney injury in patients with acute decompensated heart failure: a prospective cohort study in China. J Am Heart Assoc. (2016) 5:4035. doi: 10.1161/jaha.116.004035, PMID: 27852590 PMC5210339

[48] Section 2: Aki definition. Kidney Int Suppl. (2012) 2:19–36. doi: 10.1038/kisup.2011.32 PMC4089595 25018918

[49] Balachandran VP Gonen M Smith JJ DeMatteo RP. Nomograms in oncology: more than meets the eye. Lancet Oncol. (2015) 16:e173–80. doi: 10.1016/s1470-2045(14)71116-7, PMID: 25846097 PMC4465353

[50] Sauerbrei W Boulesteix AL Binder H. Stability investigations of multivariable regression models derived from low- and high-dimensional data. J Biopharm Stat. (2011) 21:1206–31. doi: 10.1080/10543406.2011.629890, PMID: 22023687

[51] Localio AR Goodman S. Beyond the usual prediction accuracy metrics: reporting results for clinical decision making. Ann Intern Med. (2012) 157:294–5. doi: 10.7326/0003-4819-157-4-201208210-00014, PMID: 22910942

[52] Van Calster B Vickers AJ. Calibration of risk prediction models: impact on decision-analytic performance. Med Decis Mak. (2015) 35:162–9. doi: 10.1177/0272989x14547233, PMID: 25155798

[53] Collins GS Reitsma JB Altman DG Moons KG. Transparent reporting of a multivariable prediction model for individual prognosis or diagnosis (Tripod): the Tripod statement. BMJ. (2015) 350:g7594. doi: 10.1136/bmj.g7594 25569120

